# A Critical Review on the Pharmacodynamics and Pharmacokinetics of Non-steroidal Anti-inflammatory Drugs and Opioid Drugs Used in Reptiles

**DOI:** 10.1016/j.vas.2022.100267

**Published:** 2022-08-08

**Authors:** Allison Kah Yann Ting, Vanessa Shu Yu Tay, Hui Ting Chng, Shangzhe Xie

**Affiliations:** a National University of Singapore, Department of Pharmacy, 18 Science Drive 4 Blk S4A Level 3, Singapore, 117559; b Mandai Wildlife Group, 80 Mandai Lake Road, Singapore, 729826

**Keywords:** Analgesia, Non-steroidal anti-inflammatory drugs, Opioids, Pharmacokinetics, Pharmacology, Reptiles

## Abstract

Non-steroidal anti-inflammatory drugs (NSAIDs) and opioids are analgesics used for moderate to severe pain in many animals, including reptiles. However, reptilian dosing regimens are often extrapolated from other animal species. This is not ideal as inter- and intra-species variability in physiology may result in varied drug disposition. Therefore, this critical review aims to collate data from pharmacological studies of selected NSAIDs and opioids performed in reptile and provide an analysis and discussion on the existing pharmacodynamic knowledge and pharmacokinetic data of NSAIDs and opioids use in reptiles. Additionally, key pharmacokinetic trends that may aid dosing of NSAIDs and opioids in reptiles will also be highlighted. Most of the existing reports of NSAID used in reptiles did not observe any adverse effects directly associated to the respective NSAID used, with meloxicam being the most well-studied. Despite the current absence of analgesic efficacy studies for NSAIDs in reptiles, most reports observed behavioural improvements in reptiles after NSAID treatment. Fentanyl and morphine were studied in the greatest number of reptile species with analgesic effects observed with the doses used, while adverse effects such as sedation were observed most with butorphanol use. While pharmacokinetic trends were drug- and species-specific, it was observed that clearance (CL) of drugs tended to be higher in squamates compared to chelonians. The half-life (t_1/2_) of meloxicam also appeared to be longer when dosed orally compared to other routes of drug administration. This could have been due to absorption-rate limited disposition. Although current data provided beneficial information, there is an urgent need for future research on NSAID and opioid pharmacology to ensure the safe and effective use of opioids in reptiles.

## 1 Introduction

Reptiles are divided into three main reptilian orders: *Crocodilia, Squamata* and *Chelonia*. Crocodilians include crocodiles, alligators, caimans and gharials; squamates include worm lizards, lizards and snakes; and chelonians include turtles, terrapins and tortoises (Shine, 2013). All reptiles are believed to possess anatomical components essential for pain recognition (Hawkins, 2006; Mosley, 2005). Peripheral nociceptors with Aβ- and Aδ-fibers have been identified in crotaline snakes (Liang & Terashima, 1993; Smith & Lewin, 2009) and mechanoreceptors in alligators (Kenton et al., 1971; Smith & Lewin, 2009). Additionally, a three-tiered endogenous pain control system, similar to that in mammals, has also been identified for geckos (Ten Donkelaar & de Boer-van Huizen, 1987). Neurotransmitters, essential in mammalian pain modulation, have also been identified in the Iberian wall lizard (De la Iglesia et al., 1994). Additionally, cyclooxygenase (COX) enzyme expression of ball pythons, eastern box turtles and yellow-belly geckos have been studied (Buch et al., 2018; Buch et al., 2017; Royal et al., 2012; Sadler et al., 2016). Mu- and delta-opioid receptors have also been identified in turtles (Xia & Haddad, 2001), and mu-opioid receptors in ball pythons (Kharbush et al., 2017). The presence of pain pathways and structures, as well as exposure to pain sources warrant the need for proper reptilian pain management using various drugs.

Analgesics commonly used in reptiles are non-steroidal anti-inflammatory drugs (NSAIDs) and opioids (Hawkins, 2006; Sladky & Mans, 2012). NSAIDs inhibit COX enzymes, subsequently inhibiting the conversion of arachidonic acid into inflammatory mediators such as thromboxanes and prostaglandins (Ghlichloo & Gerriets, 2021; Vane, 1971), thereby reducing inflammation and pain. Opioids exert agonistic or antagonistic effects at the mu-, delta- and kappa-opioid receptors, thereby exerting analgesic effects (Freye & Levy, 2008; James & Williams, 2020; Trescot et al., 2008). A three-step analgesic ladder was developed by the World Health Organisation to guide analgesic use in cancer (Ventafridda et al., 1985). This has been adopted in veterinary analgesic drug selection (Whiteside, 2014). For mild pain, non-opioids such as NSAIDs are used. For moderate pain, a weak opioid is used with or without a non-opioid. For severe pain, a strong opioid is used with or without a non-opioid. Generally, opioids are initiated only when NSAIDs provide insufficient analgesia. After drug selection, practitioners usually reference the Exotic Animal Formulary (EAF), a textbook of drugs and dosages used to treat exotic animals, for analgesic dose selection. Accurate dosing is imperative to provide optimal pain management, which is important in reptiles as they often do not receive analgesic treatment although they experience pain (Hawkins, 2006; Whiteside, 2014). Reptiles experience pain-induced stress which may result in detrimental effects on immune function, metabolism, hematological and serum biochemical values (Olsson & Simpson, 2017). Therefore, the provision of effective and timely analgesic treatment is paramount.

Currently, reptilian dosing is extrapolated from dosing in both closely-related and distantly-related animal species (Hawkins, 2006). However, methods for safe dose extrapolation have not been developed, and the presence of inter- and intra-species variability in physiology, and hence drug disposition (O'Malley, 2005, 2017), makes accurate dose extrapolation challenging. The physiological processes of reptiles are temperature-dependent, which can contribute to varied drug disposition among reptilian individuals, unlike endotherms with temperature-independent physiology (Mosley, 2005; Shine, 2013). In addition to temperature, other factors also contribute to varied drug disposition. For example, the renal portal system and presence of reptilian-type nephrons influence drug distribution and elimination (Holz et al., 1997b), while metabolism and feeding and foraging statuses influence drug absorption and metabolism (Secor et al., 1994). Differences in age (Reeve et al., 2015) and health status (Wilson & Bromberg, 1981) can also affect the overall drug disposition.

With such varying drug dispositions amongst reptiles, accurate dose extrapolation is likely to be challenging (Hawkins, 2006; Mosley, 2005). Additionally, there is a lack of pharmacological data to guide reptilian dosing as while many studies have been conducted to evaluate the analgesic effect of NSAIDs and opioids in mammals, few have been performed on reptiles (Mosley, 2005).

Hence, this critical review aims to collate data from pharmacological studies of NSAIDs and opioids performed in reptiles and provide an analysis and discussion on the existing pharmacodynamic knowledge and pharmacokinetic data of NSAIDs and opioids used in reptiles. Additionally, key pharmacokinetic trends that may aid dosing of NSAIDs and opioids in reptiles will also be highlighted.

## 2 Materials and Methods

A critical review was conducted through a literature search. PubMed, JSTOR, Web of Science, Scopus, ScienceDirect and Google Scholar were searched on various dates between June 2021 and May 2022 using keywords related to the topics of reptiles, physiology, pharmacology, pain management, adverse effects, NSAIDs, opioids and nociceptive tests. Detailed search terms are outlined in Appendix Table 1. References of identified papers were also searched for other papers that may be important for this review. For collation of pharmacodynamic and pharmacokinetic data, a study was included if it provided either information related to NSAIDs or opioids used in reptiles. Data extracted included: species and order of reptiles, drug name, dose, route and frequency of administration, experiment type, number and health status of subjects, peak plasma drug concentration (C_max_), time to peak plasma drug concentration (T_max_), area under the plasma concentration versus time curve from dosing to the last measured time point (AUC_0-last_), area under the plasma concentration versus time curve from dosing to infinity (AUC_0-∞_), elimination rate constant, elimination or terminal half-life (t_1/2_), bioavailability, clearance (CL), volume of distribution (V), mean residence time (MRT) adverse effects, and key conclusions from the study.

**Table 1:** List of NSAIDs commonly used in reptilian medicine and their COX selectivity (based on human studies).

**NSAID**	**COX Selectivity**	**Reference**
Meloxicam	Selective COX-2 inhibitor	(Information, 2022o)
Ketoprofen	Non-selective inhibitor	(Information, 2022c)
Tolfenamic acid	Non-selective inhibitor	(Information, 2022i)
Ketorolac	Non-selective inhibitor	(Information, 2022d)
Carprofen	Unknown	(Information, 2022a)
Flunixin	Unknown	(Information, 2022g)
Etoricoxib	Selective COX-2 inhibitor	(Information, 2022h)

## 3 General Pharmacology of NSAID and Opioid

COX is an important family of enzymes that catalyzes the rate-limiting conversion step from arachidonic acid to inflammatory prostaglandins and thromboxanes (Ghlichloo & Gerriets, 2021; Vane, 1971; Yaksh et al., 1998). COX-1 enzymes are mainly involved in gastrointestinal protection, while COX-2 enzymes in inflammation (Hawkey, 2001). NSAIDs inhibit COX enzymes, thereby reducing inflammation and pain. However, the use of COX-1 inhibitors for pain management commonly causes gastrointestinal side effects (Hawkey, 2001). Therefore, the identification of COX-2 selective inhibitor is necessary for an NSAID to provide both pain relief yet no gastrointestinal side effects. There are currently two main types of NSAIDs — COX-2 selective inhibitors and nonselective COX inhibitors. COX-1 inhibition causes the gastric and renal side effects of NSAIDs in humans (Russell, 2001), which drove the development of COX-2 selective inhibitors to minimize these side effects. Table 1 provides a non-exhaustive list of commonly used NSAIDs in reptiles.

Opioids are categorised as agonists, partial agonists, mixed agonist/antagonists or antagonists (Freye & Levy, 2008; James & Williams, 2020; Trescot et al., 2008), and exert their effect by binding to opioid receptors. Mammals possess mu-, delta- and kappa-opioid receptors (Stevens, 2009). In reptiles, past studies have found that freshwater turtles possess both mu- and delta-opioid receptors (Xia & Haddad, 2001), while ball pythons have mu-opioid receptors (Kharbush et al., 2017). Studies have also shown that mu-, delta- and kappa-opioid receptor agonists affect respiratory motor output in red-eared slider turtles (Johnson et al., 2008; Johnson et al., 2010). Additionally, many studies have determined the presence and effect of various peptides, receptors and substances in mediating pain pathways in the central nervous system of reptiles (Dores, 1982; Loeza-Alcocer et al., 2013; Reiner, 1986; Reiner, 1987). Table 2 summarises the mechanisms of action of opioids commonly used in reptilian medicine.

**Table 2:** List of opioids commonly used in reptilian medicine and their interactions with opioid receptors (based on mammalian studies).

**Opioid**	**Interaction with μ-opioid receptor**	**Interaction with κ-opioid receptor**	**Interaction with δ-opioid receptor**	**Reference**
Buprenorphine	Partial Agonist	Antagonist	Antagonist	(Information, 2022j)
Butorphanol	Partial Agonist	Partial Agonist	-	(Information, 2022m)
Fentanyl	Agonist	-	-	(Information, 2022b)
Hydromorphone	Agonist	-	-	(Information, 2022k)
Morphine	Agonist	Agonist	Agonist	(Information, 2022l)
Pethidine	Agonist	-	-	(Information, 2022e)
Tapentadol	Agonist	-	-	(Information, 2022n)
Tramadol	Agonist	-	-	(Information, 2022f)

## 4 Pharmacodynamic Data

Having understood the need for and the approaches to pain management in reptiles, data from pharmacological studies of opioids performed in reptiles were collated, analysed and discussed in the following section. Understanding the reptile-specific pharmacodynamics of NSAIDs and opioids is key to ensuring safe and efficacious administration of NSAIDs and opioids in reptiles, by allowing for optimal choice of drug and dosing.

### 4.1 NSAIDs

#### 4.1.1 Lack of COX Expression Data

Understanding COX expression in reptiles is important for predicting NSAID efficacy and toxicity in reptiles. Four studies investigating COX expression in reptiles were found and the data are summarized in Table 3. A similar trend was observed in both eastern box turtles and ball python, where significantly higher COX-1 expression was observed in inflamed than non-inflamed tissue, whereas no significant change in COX-2 expression was observed during inflammation (Royal et al., 2012; Sadler et al., 2016). However, in the eastern box turtles, COX-2 expression was higher in inflamed than non-inflamed tissue by two-fold although it was not considered significant (Royal et al., 2012). In addition, a conflicting trend was observed for COX-2 expression in yellow-belly geckos such that COX-2 gene and protein expression, and even COX-2 activity was significantly upregulated during inflammation post-amputation of their tails compared to their resting tails (Buch et al., 2018; Buch et al., 2017).

**Table 3:** Summary of Key Findings from COX Expression Studies in Reptiles.

**Species:**	**Yellow-belly Geckos**	**Yellow-belly Geckos**	**Eastern Box Turtles**	**Ball Pythons**	**Ball Pythons**
**Number of Animals**	Four groups of 6	Two groups of 20	5	6	6
**Method of Inducing Inflammation**	Amputation of tail	Amputation of tail	Differing causes of death^b^	Initially healthy Aseptic rectangular skin incision	Initially healthy Aseptic rectangular skin incision
**Tissue Type**	Tail	Tail	Muscle	Skin	Muscle
**COX-1 Protein Expression**^a^	N.R.	N.R.	Statistically significant upregulation (3-fold)	Statistically significant upregulation	Statistically significant downregulation
**COX-2 Protein Expression**^a^	Statistically significant upregulation	N.R.	Not statistically significant upregulation (but almost 2-fold)	Not statistically significant upregulation	Negligible difference
**COX-2 Gene Expression**^a^	Statistically significant upregulation	N.R.	N.R.	N.R.	N.R.
**COX-2 Activity**^a^	N.R.	Statistically significant increase	N.R.	N.R.	N.R.
**Reference**	(Buch et al., 2018)	(Buch et al., 2017)	(Royal et al., 2012)	(Sadler et al., 2016)	(Sadler et al., 2016)

a Referring to COX expression or activity during inflammation in comparison to normal conditions.

b Four suffered from head trauma, while one suffered from maggot infestation which led to amputation N.R., not reported.

Due to the limited number of COX expression studies available in reptiles and conflicting evidence, it is challenging to draw a general conclusion on how the expression of the different COX isoforms may vary during pain or inflammation in reptiles. Moreover, the current observations could be affected by potential confounders including the method of inflammation inducement, tissue type, and COX protein collection timepoints. To ensure appropriate selection of NSAIDs for specific reptile species, more mechanistic studies investigating COX expression in reptiles is required to elucidate how NSAIDs interact with the different isoforms in reptiles to elicit efficacy and side effects associated with COX-inhibition.

#### 4.1.2 Efficacy & Safety

Appendix Tables 2 and 3 summarise the range of doses and routes of administration of NSAIDs and opioids used in the various reptile species.

##### 4.1.2.1 Efficacy

Table 4 relates the COX expression data to the relevant species-specific efficacy data. Meloxicam, a coxib, showed no analgesic efficacy in ball pythons (Olesen et al., 2008), which matches the observation that COX-2 showed negligible change while COX-1 was significantly upregulated during inflammation in ball pythons (Sadler et al., 2016). In eastern box turtles, although studies showed non-statistically significant COX-2 upregulation and significant COX-1 upregulation during inflammation, COX-2 expression nonetheless doubled (Royal et al., 2012), indicating that COX-2 may still have a role in inflammation in this species, albeit less significant than COX-1. Hence, the COX data matches the signs of analgesic efficacy observed after administration of a non-selective inhibitor, ketorolac. However, it is still unknown if the NSAID COX-selectivity in reptiles is similar to humans, signaling the need for further research.

**Table 4:** Relating COX Expression Data in Reptiles to Efficacy Data.*

**NSAID Used**	**NSAID COX-selectivity in humans**	**Species (Reference)**	**Route**	**Dose**		**Observations**	**Species-specific COX upregulation during inflammation**	**Does NSAID COX-selectivity match efficacy data?**
Meloxicam (MLX)	Selective COX-2 inhibitor	Ball pythons (Olesen et al., 2008)	IM	0.3 mg/kg single dose	✗	No reduction in physiological stress and it did not appear to provide analgesia to the pythons.	COX-1 significantly upregulated (Sadler et al., 2016). COX-2 showed negligible change (Sadler et al., 2016).	Yes
Ketorolac (KT)	Non-selective COX inhibitor	Eastern Box Turtles (Cerreta et al., 2019)	IM	0.25 mg/kg single dose	**≈**	Improvement in appetite and activity level during rehabilitation. Note: no other analgesic agents were administered concurrently.	COX-1 significantly upregulated (Royal et al., 2012). COX-2 showed non-statistically significant upregulation (but almost 2-fold increase) (Royal et al., 2012).	Yes
		Eastern Box Turtles (Henson & Lewbart, 1998)	IM	N.R. single dose	✓	KT-treated turtles showed faster recovery, began eating and behaving normally earlier than the control group.		Yes

⁎ COX selectivity of NSAIDs in humans assumed to be similar to that in reptiles; IM, intramuscular administration; COX, cyclooxygenase; ✗, no sign of analgesic efficacy; **≈**, possible sign of analgesia but no direct indication of efficacy; ✓, clear indication of analgesic efficacy observed; N.R., not reported.

Table 5 presents a non-exhaustive summary of observations indicating possible analgesic efficacy of NSAIDs in reptiles. Most of the reported NSAIDs used only showed probable indicators of analgesic efficacy. Though meloxicam administration showed pain-related improvements in multiple cases, the direct association of meloxicam to true analgesic efficacy in reptiles remains unclear. There was one exception from a conference proceeding by Hensen et al. that showed a direct association between NSAID treatment and analgesic efficacy (Henson & Lewbart, 1998). Unlike the other studies, this study showed significantly quicker improvement in the ketorolac-treated eastern box turtles and yellow-bellied sliders conditions based on their time taken to start eating post-surgery and their activity score when compared to the control group (Henson & Lewbart, 1998). Henceforth, future NSAID efficacy studies should ensure a comparison against a control group is made and confounders are accounted for. Additionally, future NSAID efficacy studies should utilize validated nociceptive methods such as formalin tests, instead of using subjective methods like through the observation of the subjects’ improvements in behaviour, as validated nociceptive methods are more accurate and hence useful for assessing analgesic efficacy.

**Table 5:** Summary of Efficacy-related Information of NSAIDs in Reptiles.

**Dosage**	**Dose found in EAF**	**Species**	**Reason for Treatment**		**Efficacy**	**Efficacy Assessment Tool Used**	**Reference**
**MELOXICAM (MLX)**^c^							
PO 10.5 mg single dose	N	Komodo dragon	Initial treatment for severe osteoarthritis.	**≈**	Improvements in ambulation, posture, and strength.	N.R.	(Wolfe et al., 2015)
IM 0.3 mg/kg single dose	Y	Ball pythons	Preoperative treatment before catheterization.	✗	No reduction in physiological stress and did not appear to provide analgesia.	Observed heart rate, blood pressure, mean plasma epinephrine and cortisol concentration	(Olesen et al., 2008)
IM 0.2 mg/kg Q24h^a^	Y	Bearded dragon	Postoperative treatment after external coaptation and suturing for fractured mandible.	**≈**	Resumed normal behaviour within a week. No signs indicating discomfort.	N.R.	(McDermott, 2021)
IM 0.2 mg/kg Q48h x 14d	Y	Bearded Dragon	Administered postoperatively after surgery for fractured mandible.	**≈**	Lizard recovered well, showing no indications of discomfort.	N.R.	(McDermott, 2021)
**FLUNIXIN (FNX)**^d^							
IM 0.5 mg/kg Q24h^a^	Y	Monitor Lizard	Used as anti-inflammatory treatment for severe fibrin-necrotic enteritis, together with antimicrobials.	**≈**	Lizard recovered well.	N.R.	(Seixas et al., 2014)
**Dosage**	**Dose found in EAF**	**Species (Reference)**	**Reason for Treatment**		**Observations**	**Efficacy Assessment Tool Used**	**Ref-erence**
**KETOROLAC (KT)**^b^							
IM 0.25 mg/kg single dose	N	Eastern Box Turtles	Analgesia for traumatic injury.	**≈**	Improvement in appetite and activity level during rehabilitation.	N.R.	(Cerreta et al., 2019)
N.R IM single dose	N	Eastern Box Turtles & Yellow-bellied Sliders	Treatment of minor shell fractures in turtles, with no other injuries or conditions.	✓	KT-treated turtles showed faster recovery, began eating and behaving normally earlier than the control group.	Monitored heart rate, time taken to return to normal feeding and normal activity level.	(Henson & Lewbart, 1998)
IM 0.25 mg/kg Q24h x 5d	N	Loggerhead Sea Turtle	Administered postoperatively as analgesic after severe skull fracture repair.	**≈**	After one month of intensive care, wound irrigation and KT course, the turtle began eating normally and its condition improved.	N.R.	(Lewbart et al., 2001)
**ETORICOXIB**							
PO 25 mg/kg Q24h^a^	N	Yellow-bellied Gecko	To study effect of COX-2 inhibition on Wnt/β-Catenin signalling. An activity assay of COX-2 was conducted, comparing activity between an amputated tail versus resting tail.	**≈**	Etoricoxib significantly inhibited COX-2 in the wound epidermis and blastema. COX-2 activity was also observed to be significantly higher in wound epidermis compared to resting tail.	N.R.	(Buch et al., 2017)

IM, intramuscular; PO, oral administration; q, every (e.g. “q24h” means “every 24 hours"); d, days (e.g. “10d” means “10 days”); N.R., not reported; **≈**, possible sign of analgesia but no direct indication of efficacy; ✓, analgesic efficacy observed; N, dosing regimen not found in the Exotic Animal Formulary (a textbook of drugs and dosages used to treat exotic animals); Y, dosing regimen found in the Exotic Animal Formulary (a textbook of drugs and dosages used to treat exotic animals).

a Unspecified duration of treatment

b non-selective COX inhibitor in humans

c COX-2 selective inhibitors in humans

d undetermined COX selectivity in humans

Several cases showed efficacy of non-pharmacological methods of pain relief. For example, physical therapy was shown to be a viable option in Komodo dragons as part of osteoarthritis treatment (Wolfe et al., 2015) and McDermott et al. supported the use of external coaptation to minimize movement in injured bearded dragons (McDermott, 2021). Thus, the use of non-pharmacological analgesic methods in conjunction with pharmacological therapy should be considered favorably in reptiles by clinicians. However, it should be noted that many of these studies had small sample sizes and hence, the results should be interpreted with caution.

Overall, more analgesic efficacy studies in NSAIDs should be carried out in future utilizing more accurate assessment tools like nociceptive assays, instead of solely observing improvements in behaviour.

##### 4.1.2.2 Safety

The current NSAID toxicity and safety concerns in reptiles are largely similar to that in humans and mammals. Concerns have been raised about reptiles having higher risk of NSAID-related gastrointestinal adverse effects due to their infrequent and inconsistent feeding habits especially when receiving NSAIDs on an empty stomach (Lai et al., 2015). This risk is further exacerbated when repeated NSAID doses are administered. Furthermore, with the presence of significantly fewer nephrons in reptiles compared to mammals, they might theoretically be more prone to nephrotoxic adverse effects (Lai et al., 2015). However, we are unable to validate these concerns yet due to our limited understanding of NSAID toxicity and safety in reptiles.

Most reports of NSAID use in reptiles currently either do not observe any adverse effects or do not report about NSAID side effects. A summary of the several cases that did report possible adverse effects and bioaccumulation information of NSAIDs in reptiles is provided in Table 6. This summary serves to offer a comprehensive overview of all adverse effects reported in reptiles that may be associated with NSAID use and to highlight the cases where no adverse effects were observed. Since most of the adverse effects reported could not be directly associated with NSAID use alone, further toxicity and safety studies are required.

**Table 6:** Summary of Toxicity- and Safety-Related Information of NSAIDs in Reptiles.

**Dosage**	**Dose found in EAF**	**Species**	**Reason for Treatment**		**Observations**	**Ref-erence**
**MELOXICAM (MLX)**						
PO, IV 0.2 mg/kg^c^ single dose	Y	Green Iguanas	Pharmacokinetic study on MLX in the iguanas.	**≈**	Well-tolerated, no changes in weight, feeding or evidence of vomiting. No abnormalities found in stomach, liver, and kidneys.	(Divers et al., 2010)
PO 1 or 5 mg/kg^d^ Q24h x 12d	N			✗	High uric acid and total protein concentrations, high total WBC, heterophil, lymphocyte, and monocyte counts. Note: Uric acid only exceeded normal range when given 5 mg/kg multiple doses. Two iguanas formed a single small bacterial granuloma in the pancreas or kidney – deemed clinically unimportant by authors. Histology of stomach, liver and kidneys were unremarkable.	
IM 0.2 mg/kg^c^ Q24h x 10d	Y	Green Iguanas	Study on the effect of MLX on the blood profiles of the iguanas.	**≈**	Lowered plasma calcium (Ca) levels, haemoglobin, and packed cell volume (PCV) observed. Increase in mean alanine aminotransferase (ALT) levels.^a^ All blood parameters after the 10 days were within normal ranges and with no negative impact on their health.	(Trnkova et al., 2007)
IM 0.2 mg/kg^c^ Q48h x 14d	Y	Bearded Dragon	Postoperative analgesic for mandible fracture.	**≈**	No stomatitis observed.^b^	(McDermott, 2021)
**MELOXICAM (MLX)**						
IM, IV 0.1 mg/kg^c^ single dose	Y	Loggerhead Sea Turtles	Pharmacokinetic study on MLX in the turtles.	**≈**	No adverse effects observed. ^b^	(Lai et al., 2015)
IM, IV 0.2 mg/kg^c^ single dose	Y	Red-eared Sliders	Pharmacokinetic study on MLX in the sliders.	**≈**		(Uney et al., 2016)
SC 1 mg/kg^d^ single dose	N	Kemp's Ridley, Loggerhead and Green Sea Turtles	Pharmacokinetic study on MLX in the turtles.^f^	**≈**		(Norton et al., 2021)
**KETOPROFEN (KTP)**						
IM, IV^e^ 2 mg/kg^c^ single dose	Y	Loggerhead Sea Turtles	Pharmacokinetic study on KTP in the turtles.	**≈**	Anemia observed in one of the 20 turtles but it maintained normal behaviour; speculated to be caused by repeated blood withdrawals causing subcutaneous hematomas. Anemia went away without treatment within following week.	(Thompson et al., 2018)^a^
IM 2 mg/kg^c^ Q24h x 3d	Y			**≈**	No bioaccumulation occurs with repeated dosing at this dose.	
**KETOPROFEN (KTP)**						
IM 2 mg/kg^y^ Q24h x 5d	Y	Loggerhead Sea Turtles	Safety study on repeated KTP IM doses in the turtles.	**≈**	No abnormalities detected in weight, plasma biochemistry and haematology data. Behaviour and appetite remained normal. No bioaccumulation observed. Note: Other potential side effects were not studied.	(Harms et al., 2021)
**CARPROFEN (CRP)**						
IM 2 mg/kg^y^ Q24hx 10d	Y	Green Iguanas	Study on the effect of CRP on the blood profiles of the iguanas.	**≈**	Higher mean ALT and aspartate aminotransferase (AST) levels in CRP-treated iguanas compared to MLX- treated and control iguanas. Reduced haemoglobin and PCV, with increase in percentage azurophils observed.^g^ All parameter levels were still within normal ranges, with no negative health impact.	(Trnkova et al., 2007)
**FLUNIXIN (FNX)**						
IM 1^h, i^ mg/kg^c^	Y	Green Sea Turtles	Treatment for turtles undergoing extensive fibro-papilloma removal surgery.	✗	Associated with fatal gastroenteritis.	(Wyneken et al., 2006)
**KETOROLAC (KT)**^j^						
IM 0.25 mg/kg single dose	N	Loggerhead Sea Turtles	Pharmacokinetic study on KT in the turtles.^f^	**≈**	No significant adverse effects observed. One turtle acted abnormally 18 hours post-administration, it floated immobile on surface but returned to normal behaviour by the end of the study. Postulated reason given by the author was stress caused by handling.	(Gregory et al., 2021)^f^
IM 0.25 mg/kg single dose	N	Eastern Box Turtles	Pharmacokinetic study on KT in the turtles.	**≈**	No significant adverse effects observed ^b^	(Cerreta et al., 2019)
**TOLFENAMIC ACID (TA)**^j^						
IV, IM 4 mg/kg single dose	N	Green Sea Turtles	Pharmacokinetic study of TA in healthy green sea turtles.	**≈**	No adverse effects, behavioural or health alterations were observed.^b^	(Raweewan et al., 2020a)
IV, IM 4 mg/kg single dose	N	Hawksbill turtles	Pharmacokinetic study of TA in healthy hawksbill turtles.	**≈**	No adverse effects, behavioural or health alterations were observed during or after the study for 1 month.^b^	(Raweewan et al., 2020b)
IV, IM 2 mg/kg single dose	N	Red-eared sliders	Pharmacokinetic study in healthy red-eared sliders.	**≈**	No local or systemic adverse effects were observed in any turtle.^b^	(Corum et al., 2019)
IV 4 mg/kg single dose	N		Pharmacokinetic study in healthy red-eared sliders.			

^a^ Changes in Ca, haemoglobin, PCV and ALT also observed in control, so changes might not be related to meloxicam administration. ^b^ Specifically stated in study or report that no adverse effects were observed at the given doses but no further details given. ^c^ adheres to EAF recommended dosing. ^d^ higher than EAF recommended dosing. ^e^ IV administration of ketoprofen is not in EAF. ^f^ Juvenile loggerhead sea turtles were used in this study. ^g^ Reduction in haemoglobin and PCV also seen in control, so these changes may not be due to CRP. ^h^ Unspecified dosing frequency. ^i^ Unspecified duration of treatment. ^j^ NSAID not listed in EAF aJuvenile loggerhead sea turtles were used in this study. IV, intravenous; IM, intramuscular; SC, subcutaneous; PO, per oral; q, every (e.g. “q24h” means “every 24 hours"); d, days (e.g. “10d” means “10 days”); N.A., not applicable; ≈, no clinically significant adverse effects observed; ✗, clinically significant adverse effects were observed; Y, dosing regimen found in the Exotic Animal Formulary (a textbook of drugs and dosages used to treat exotic animals); N, dosing regimen not found in the Exotic Animal Formulary (a textbook of drugs and dosages used to treat exotic animals).

Meloxicam is the most extensively studied NSAID, with almost no direct association between its use and adverse effects being reported yet, except for a study in green iguanas where repeated high doses of 1 or 5 mg/kg was administered. Uric acid levels exceeded the normal range when multiple doses of 5 mg/kg meloxicam were administered, whereas no adverse effects were observed at 0.2 mg/kg dose in the two green iguana studies (Divers et al., 2010) (Table 6). Therefore, to err on the side of safety, meloxicam doses exceeding 0.2 mg/kg should be avoided in green iguanas. For the other species reported, no adverse effects were observed at the respective doses and administration routes. Hence, it is relatively safe for future researchers to use these doses.

A dose of 2 mg/kg of ketoprofen and repeated doses of 2 mg/kg Q24h for five days was also shown to be safe in the loggerhead sea turtles study by comparing blood parameters with a control (Thompson et al., 2018) (Table 6). However, other potential side effects that were not studied cannot be completely ruled out. For flunixin, 1 mg/kg should be avoided in green sea turtles due to reported association with fatal gastroenteritis (Wyneken et al., 2006) (Table 6). A lower dose of flunixin should be used in green sea turtles with close monitoring. For carprofen, ketorolac and tolfenamic acid, administration of the doses shown in Table 6 can be given for future research since no significant adverse effects were reported.

There were two studies in Table 6 that were conducted in juvenile subjects and since juvenile and adult animals may differ physiologically, the safety profiles may also differ. However, though these two studies did not report any significant adverse effects, anemia was observed in one turtle for one study (Thompson et al., 2018), and abnormal behaviour was observed in a turtle for the other (Gregory et al., 2021). Regarding the abnormal behaviour of one of the turtles 18 hours after ketorolac administration (Gregory et al., 2021), while the author attributed this behaviour to stress due to handling, we cannot rule out the possibility that it was caused by the administration of ketorolac. Therefore, future administration of the respective NSAIDs at these doses should be done with caution.

Overall, researchers and clinicians should be cautious when exploring higher doses than reported (Table 6) or when administering repeated NSAID doses, particularly in reptiles in a dehydrated state, with renal or liver impairment, or with gastrointestinal conditions. Although it is relatively safer to use the indicated doses and administration routes in Table 6 that shows no adverse effects, due to the small number of NSAID studies in reptiles, adverse effects at these doses cannot be ruled out completely. Additionally, there are currently only NSAID toxicity and safety data in chelonians and squamates, but none in crocodilians. Therefore, researchers and clinicians must exercise extra caution especially in NSAID administration to crocodilians.

### 4.2 Opioids

#### 4.2.1 Lack of Opioid Receptor Expression Data

Data on opioid receptor expression in reptiles is limited with the only information available being the identification of mu- and delta-opioid receptors in turtles (Xia & Haddad, 2001), mu-opioid receptors in ball pythons (Kharbush et al., 2017), as well as data that mu-, delta-, and kappa-opioid receptor agonists affect respiration in red-eared slider turtles (Johnson et al., 2008; Johnson et al., 2010).

#### 4.2.2 Efficacy & Safety

The effect of eight different opioids, used in 12 reptilian species (1 crocodilian, 6 squamates, and 5 chelonians) were evaluated in 21 studies (Table 7). The studies were mainly performed in squamates and chelonians as they are generally smaller in size compared to crocodilians and are thus easier to handle. Of the chelonian species studied, red-eared slider turtles were most commonly studied as they are the more common species kept as pets.

**Table 7:** Summary of the efficacy and adverse effects observed in studies of buprenorphine, butorphanol, fentanyl, hydromorphone, morphine, pethidine, tapentadol and tramadol on reptiles.

**Dosage**	**Dose found in EAF**		**Species**				**Method of Pain Infliction**			**Efficacy**					**Adverse Effects**			**Ref-erence**
**BUPRENORPHINE**																		
**Squamata**																		
IM 0.02mg/kg	N		Green iguana				Electro-stimulation			✗	No significant difference in body movement response scores				None observed			(Greenacre et al., 2006)
IM 0.1mg/kg	N		Green iguana				Electro-stimulation			✗	No significant difference in body movement response scores				None observed			(Greenacre et al., 2006)
**Chelonia**																		
SC 0.02mg/kg	N	Red-eared slider turtle				-				✗^a^	43% (forelimb administration) and 21% (hindlimb administration) of animals tested maintained target plasma level of >1 ng/ml at 24 hr				GI stasis (resolved over 5 days)			(Kummrow et al., 2008)
SC 0.05mg/kg	N	Red-eared slider turtle				-				✓^b^	85% of animals tested maintained target plasma level of >1 ng/ml at 24 hr				GI stasis (resolved over 5 days)			(Kummrow et al., 2008)
SC 0.1mg/kg	N	Red-eared slider turtle				Thermal stimulus				✗	Lack of increase in hindlimb withdrawal latencies hence no apparent analgesia				Not reported			(Mans et al., 2012)
SC 0.2mg/kg	Y	Red-eared slider turtle				Thermal stimulus				✗	Lack of increase in hindlimb withdrawal latencies hence no apparent analgesia				Not reported			(Mans et al., 2012)
SC 1mg/kg	N	Red-eared slider turtle				Thermal stimulus				✗	Lack of increase in hindlimb withdrawal latencies hence no apparent analgesia				Not reported			(Mans et al., 2012)
**BUTORPHANOL**																		
**Squamata**																		
SC 2mg/kg	N	Bearded dragon					Thermal stimulus			✗	Withdrawal latencies almost identical to baseline at 2-24hr				Not reported			(Sladky et al., 2008)
		Corn snake					Thermal stimulus			✗	Withdrawal latencies not altered from baseline at 2-24hr				Not reported			(Sladky et al., 2008)
SC 20mg/kg	Y	Bearded dragon					Thermal stimulus			✗	No significant increase in withdrawal latencies from baseline at 2-24hr				Not reported			(Sladky et al., 2008)
		Corn snake					Thermal stimulus			✓	Significant increase in withdrawal latencies at 2-24hr				Not reported			(Sladky et al., 2008)
IM 0.4mg/kg	Y	Green iguana					Electro-stimulation			✗	No significant difference in body movement response scores				None observed			(Greenacre et al., 2006)
IM 1.5mg/kg	N	Green iguana					Electro-stimulation			✓	Significantly lower body movement response scores hence provides analgesia				None observed			(Greenacre et al., 2006)
IM 4mg/kg	N	Green iguana					Electro-stimulation			✗	No significant difference in body movement response scores				None observed			(Greenacre et al., 2006)
IM 5mg/kg	N	Ball python					Surgery			✗	No effect on postoperative physiologic variables				Not reported			(Olesen et al., 2008)
		Tegus					Thermal stimulus			✗	No significant difference in withdrawal latencies from baseline				Not reported			(Leal et al., 2017)
IM 8mg/kg	N		Green iguana				Electrostimulation			✓	Significantly lower body movement response scores hence provides analgesia				Delayed righting reflex observed in one Green iguana		(Greenacre et al., 2006)	
IM 10mg/kg	N		Ball python				Capsaicin injection			✗	Failed to reduce tachycardia caused by capsaicin				Heavy sedation, reduced muscle tone		(Williams et al., 2016)	
			Tegus				Thermal stimulus			✗	No significant difference in withdrawal latencies from baseline				Not reported		(Leal et al., 2017)	
**Chelonia**																		
SC 2.8mg/kg	N				Red-eared slider turtle		Thermal stimulus			✗	No difference in withdrawal latencies from baseline; Significant time-dependent effect at 2-24hr				Not reported		(Sladky et al., 2007)	
SC 20mg/kg	Y				Red-eared slider turtle		Thermal stimulus			✓	Elevated withdrawal latencies up to 6hr				None observed		(Kinney et al., 2011)	
SC 28mg/kg	N				Red-eared slider turtle		Thermal stimulus			✗	No difference in withdrawal latencies from baseline; Significant time-dependent effect at 2-24hr				Short-term respiratory depression		(Sladky et al., 2007)	
**FENTANYL**																		
**Squamata**																		
TD 2.5ug/h	N				Prehensile-tailed skink		-			✓	Concentrations measured within human analgesic range by 36h				None observed		(Gamble, 2008)	
TD 3ug/h	N				Ball python		Thermal stimulus			✗	Withdrawal latencies similar to baseline at 3-48hr				Not reported		(Kharbush et al., 2017)	
TD 12ug/h	N				Ball python		Thermal stimulus			✓	Withdrawal latencies similar to baseline at 3-48hr but high plasma concentrations up to 48hr were detected				Depressed breathing frequency		(Kharbush et al., 2017)	
							-			✓	Theoretical analgesic plasma concentration of ≥1ng/ml achieved within ≤4h and maintained for 7-day study duration				None observed		(Darrow et al., 2016)	
**Chelonia**																		
SC 0.05mg/kg	N			Black-bellied slider turtle			Limb pinch test		✓		All animals tested showed signs of analgesia within 10min; 80% showed total absence of response to nociceptive stimulus lasting about 15min				None observed		(Kaminishi et al., 2019)	
				Red-eared slider turtle			Limb pinch test		✓		90% of animals tested showed signs of analgesia within 10min, remaining 10% showed signs of analgesia within 20min; 80% showed total absence of response to nociceptive stimulus lasting about 15min				None observed		(Kaminishi et al., 2019)	
**HYDROMORPHONE**																		
**Squamata**																		
SC 0.5mg/kg	Y			Bearded dragon			-			✓		Theoretical analgesic plasma concentration of >4ng/ml detectable up to 24hr		None observed			(Hawkins et al., 2019)	
SC 1mg/kg	Y			Bearded dragon			-			✓		Theoretical analgesic plasma concentration of >4ng/ml detectable up to 24hr		Mild sedation			(Hawkins et al., 2019)	
***Chelonia***																		
SC 0.5mg/kg	Y			Red-eared slider turtle			Thermal stimulus			✓		Significant increase in hindlimb withdrawal latencies for up to 24hr hence provides thermal analgesia		None observed			(Mans et al., 2012)	
							-			✓		Theoretical analgesic plasma concentration of >4ng/ml detectable at 12hr		None observed			(Hawkins et al., 2019)	
SC 1mg/kg	Y			Red-eared slider turtle			-			✓		Theoretical analgesic plasma concentration of >4ng/ml detectable at 12hr		None observed			(Hawkins et al., 2019)	
**MORPHINE**																		
**Crocodilia**																		
IP 0.05mg/kg	N			Crocodile			Hot plate test			✓		Statistically significant increase in response latencies of 'lifting foot' and 'escape' pain-related behaviours		Not reported		(Kanui & Hole, 1992)		
IP 0.1mg/kg	N			Crocodile			Hot plate test			✓		Statistically significant increase in response latencies of 'lifting foot' and 'escape' pain-related behaviours		Not reported		(Kanui & Hole, 1992)		
IP 0.2mg/kg	N			Crocodile			Hot plate test			✓		Statistically significant increase in response latencies of 'lifting foot' and 'escape' pain-related behaviours		Not reported		(Kanui & Hole, 1992)		
IP 0.3mg/kg	N			Crocodile			Hot plate test			✓		Statistically significant increase in response latencies of 'lifting foot' and 'escape' pain-related behaviours; Maximal response latencies at this dose			Not reported	(Kanui & Hole, 1992)		
IP 1mg/kg	N			Crocodile			Hot plate test			✓		Statistically significant increase in response latencies of 'lifting foot' and 'escape' pain-related behaviours			Not reported	(Kanui & Hole, 1992)		
**Squamata**																		
SC 1mg/kg	N			Bearded dragon			Thermal stimulus			✗		No significant increase in withdrawal latencies from baseline at 2-24hr		Not reported		(Sladky et al., 2008)		
				Corn snake			Thermal stimulus			✗		No significant increase in withdrawal latencies from baseline at 2-24hr		Not reported		(Sladky et al., 2008)		
SC 5mg/kg	N			Bearded dragon			Thermal stimulus			✗		No significant increase in withdrawal latencies from baseline at 2-24hr		Not reported		(Sladky et al., 2008)		
				Corn snake			Thermal stimulus			✗		No significant increase in withdrawal latencies from baseline at 2-24hr		Not reported		(Sladky et al., 2008)		
SC 10mg/kg	N			Bearded dragon			Thermal stimulus			✓		Significant increase in withdrawal latencies at 2-24hr		Not reported		(Sladky et al., 2008)		
				Corn snake			Thermal stimulus			✗		No significant increase in withdrawal latencies from baseline at 2-24hr		Not reported		(Sladky et al., 2008)		
SC 20mg/kg	N			Bearded dragon			Thermal stimulus			✓		Significant increase in withdrawal latencies at 2-24hr		Not reported		(Sladky et al., 2008)		
				Corn snake			Thermal stimulus			✗		No significant increase in withdrawal latencies from baseline at 2-24hr		Not reported		(Sladky et al., 2008)		
SC 40mg/kg	N			Corn snake			Thermal stimulus			✗		No significant increase in withdrawal latencies from baseline at 2-24hr		Not reported		(Sladky et al., 2008)		
IM 0.4mg/kg	N			Green iguana			Electrostimulation			✗		No significant difference in body movement response scores		None observed		(Greenacre et al., 2006)		
IM 1mg/kg	Y			Green iguana			Electrostimulation			✓		Significantly lower body movement response scores hence provides analgesia		None observed		(Greenacre et al., 2006)		
IM 5mg/kg	N			Tegus			Thermal stimulus			✓		Significantly higher withdrawal latencies for at least 12hr		Not reported		(Leal et al., 2017)		
IM 10mg/kg	Y			Ball python			Capsaicin injection			✗		Failed to reduce tachycardia caused by capsaicin		None observed		(Williams et al., 2016)		
				Tegus			Thermal stimulus			✓		Significantly higher withdrawal latencies for at least 12hr		Not reported		(Leal et al., 2017)		
**Chelonia**																		
SC 1.5mg/kg	Y			Red-eared slider turtle			Thermal stimulus			✓			Significant increase in withdrawal latencies; Significant drug effect at 0-24hr	Long-lasting respiratory depression		(Sladky et al., 2007)		
SC 2mg/kg	Y			Red-eared slider turtle			Thermal stimulus			✓			Elevated withdrawal latencies beyond 6hr	Depressed breathing and feeding		(Kinney et al., 2011)		
SC 6.5mg/kg	Y			Red-eared slider turtle			Thermal stimulus			✓			Significant increase in withdrawal latencies; Significant drug effect at 0-24hr	Not reported		(Sladky et al., 2007)		
ICo 5mg/kg	N			Speke's hinged tortoise			Formalin test			✗			No statistically significant decrease in time spent in nocifensive behaviour	Not reported		(Wambugu et al., 2010)		
ICo 7.5mg/kg	N			Speke's hinged tortoise			Formalin test			✓			Statistically significant decrease in time spent in nocifensive behaviour hence provides analgesia	Not reported		(Wambugu et al., 2010)		
ICo 10mg/kg	N			Speke's hinged tortoise			Formalin test			✓			Statistically significant decrease in time spent in nocifensive behaviour hence provides analgesia	Not reported		(Wambugu et al., 2010)		
ICo 20mg/kg	N			Speke's hinged tortoise			Formalin test			✓			Statistically significant decrease in time spent in nocifensive behaviour hence provides analgesia	Not reported		(Wambugu et al., 2010)		
**PETHIDINE**																		
**Crocodilia**																		
IP 1mg/kg	N			Crocodile			Hot plate test			✓			Statistically significant increase in response latencies of 'lifting foot' and 'escape' pain-related behaviours	Not reported		(Kanui & Hole, 1992)		
IP 2mg/kg	N			Crocodile			Hot plate test			✓			Statistically significant increase in response latencies of 'lifting foot' and 'escape' pain-related behaviours; Maximal response latencies at this dose	Not reported		(Kanui & Hole, 1992)		
IP 4mg/kg	N			Crocodile			Hot plate test			✓			Statistically significant increase in response latencies of 'lifting foot' and 'escape' pain-related behaviours; Maximal response latencies at this dose	Not reported		(Kanui & Hole, 1992)		
IP 6mg/kg	N			Crocodile			Hot plate test			✓			Statistically significant increase in response latencies of 'lifting foot' and 'escape' pain-related behaviours	Not reported		(Kanui & Hole, 1992)		
IP 8mg/kg	N			Crocodile			Hot plate test			✓			Statistically significant increase in response latencies of 'lifting foot' and 'escape' pain-related behaviours	Not reported		(Kanui & Hole, 1992)		
**Chelonia**																		
ICo 10mg/kg	N			Speke's hinged tortoise			Formalin test			✗			No statistically significant decrease in time spent in nocifensive behaviour	Not reported		(Wambugu et al., 2010)		
ICo 20mg/kg	N			Speke's hinged tortoise			Formalin test			✓			Statistically significant decrease in time spent in nocifensive behaviour hence provides analgesia	Not reported		(Wambugu et al., 2010)		
ICo 50mg/kg	N			Speke's hinged tortoise			Formalin test			✓			Statistically significant decrease in time spent in nocifensive behaviour hence provides analgesia	Not reported		(Wambugu et al., 2010)		
**TAPENTADOL**																		
**Chelonia**																		
IM 5mg/kg	N			Red-eared slider turtle			Thermal stimulus			✓			Significant increase in hindlimb withdrawal latencies for up to 10hr hence provides analgesia	Sedation, Unresponsive to external stimuli, Flaccid limbs and necks (resolved within 2h)		(Giorgi et al., 2014)		
				Yellow-bellied slider turtle			Thermal stimulus			✓			Significant increase in hindlimb withdrawal latencies for up to 10hr hence provides analgesia	Sedation, Unresponsive to external stimuli, Flaccid limbs and necks (resolved within 2h)		(Giorgi et al., 2015a)		
**TRAMADOL**																		
**Chelonia**																		
PO 1mg/kg	N			Red-eared slider turtle			Thermal stimulus			✗			Significant increase in withdrawal latencies at 12hr, but no significant drug effect	Not reported		(Baker et al., 2011)		
PO 5mg/kg	Y			Logger-head sea turtle				-		✓			Target plasma level of ≥100ng/ml maintained for 48hr	None observed		(Norton et al., 2015)		
				Red-eared slider turtle				Thermal stimulus		✓			Significant increase in withdrawal latencies at 12 and 24 hr hence provides thermal analgesia	Respiratory depression		(Baker et al., 2011)		
PO 10mg/kg	Y			Logger-head sea turtle				-		✓			Target plasma level of ≥100ng/ml maintained for 72hr	None observed		(Norton et al., 2015)		
				Red-eared slider turtle				Thermal stimulus		✓			Significant increase in withdrawal latencies for 6-96hr hence provides thermal analgesia	Respiratory depression		(Baker et al., 2011)		
PO 25mg/kg	N			Red-eared slider turtle				Thermal stimulus		✓			Significant increase in withdrawal latencies for 6-96hr hence provides thermal analgesia	Severe respiratory depression, Flaccid limbs and necks		(Baker et al., 2011)		
SC 10mg/kg	Y			Red-eared slider turtle				Thermal stimulus		✓			Significant drug effects compared with control treatment	Not reported		(Baker et al., 2011)		
SC 25mg/kg	N			Red-eared slider turtle				Thermal stimulus		✓			Significant drug effects compared with control treatment	Not reported		(Baker et al., 2011)		
IM 10mg/kg	Y			Yellow-bellied slider turtle				Thermal stimulus		✓			Significant increase in hindlimb withdrawal latencies over 0.5-48hr (forelimb administration) and 8-48h (hindlimb administration)	None observed		(Giorgi et al., 2015b)		
**TRAMADOL (M1)**																		
**Chelonia**																		
PO 5mg/kg	Y			Logger-head sea turtle				-		✓			Target plasma level of ≥100ng/ml maintained for 48hr	None observed		(Norton et al., 2015)		
PO 10mg/kg	Y			Logger-head sea turtle				-		✓			Target plasma level of ≥100ng/ml maintained for 72hr	None observed		(Norton et al., 2015)		
IM 10mg/kg	Y			Yellow-bellied slider turtle				Thermal stimulus		✓			Detected in plasma for up to 96hr	None observed		(Giorgi et al., 2015b)		

^a^, percentage of animals that maintained target plasma level of 1ng/ml at 24hr was far below the desired percentage of 90%. ^b^, percentage of animals that maintained target plasma level of 1ng/ml at 24hr was close to the desired percentage of 90. IM, intramuscular injection; SC, subcutaneous injection; IP, intraperitoneal injection; ICo, intracoelomic injection; TD, transdermal patch; PO, by mouth; Y, dosing regimen found in EAF; N, dosing regimn not found in EAF; ✓, analgesic efficacy observed; ✗, no analgesic efficacy observed.

The eight opioids studied were buprenorphine, butorphanol, fentanyl, hydromorphone, morphine, pethidine, tapentadol, and tramadol. Butorphanol and morphine were the most widely studied in different species, with six and seven species, each.

Table 7 highlights the safety and efficacy data of opioids tested in reptilian species, with an overview summarised in Appendix Tables 4 and 5. Firstly, to determine analgesic efficacy, pain was inflicted via various validated methods such as capsaicin or formalin injections, electrostimulation, thermal stimulation, surgery and limb pinches. Analgesia was then evaluated through objective measures such as change in heart rate, duration of limb retraction, body movement response scores and thermal withdrawal latencies. Analgesia was distinguished from sedation with sedation being determined based on muscle tone and extent of voluntary movement and righting reflex. The opioids with the most pharmacological data to support their use are fentanyl and morphine, which provided analgesia to mainly squamates and chelonians. Although tested in fewer species, butorphanol and hydromorphone provided analgesia to both squamates and chelonians, while buprenorphine provided analgesia only to chelonians. However, it is important to note that these tests mainly evaluate acute pain, with no tests evaluating chronic pain. This limits the value of opioid efficacy data as treatment of chronic pain is of uppermost clinical interest.

With regards to side-effects, the opioids with the most evidence of adverse effects are butorphanol and tapentadol, which exhibited adverse effects such as sedation mainly in squamates and chelonians, respectively. Buprenorphine and morphine exhibited adverse effects mainly in chelonians, while fentanyl and hydromorphone exhibited adverse effects mainly in squamates. As tapentadol and tramadol were only tested in chelonians, and pethidine was only tested in crocodilians and chelonians, we are unsure if these opioids provide analgesia or exhibit toxicity in squamates. Additionally, as only morphine and pethidine were tested in crocodiles, we are unsure of the efficacy and safety of other opioids in crocodilians.

Not all studies assessed efficacy via nociceptive tests as some evaluated efficacy based on whether plasma drug concentrations reached human analgesic plasma levels. This is a potential limitation as analgesic plasma concentrations in humans may differ from that in reptiles. Hence, it is challenging to conclude if the opioid is truly effective in alleviating pain for the reptile tested.

### 4.3 Critical Analysis on the Design of Pharmacodynamic Studies

Nociceptive tests are dependent on physical signs of pain, such as withdrawal reflexes, aggression and verbalization (Carter & Shieh, 2015), but these signs are challenging to assess in reptiles. In the pharmacological studies obtained from our literature review, nociception was assessed via various methods: capsaicin injection, electrostimulation, formalin test, thermal stimulus test, surgery, hot plate test and limb pinch test. Thermal stimulus tests were most commonly adopted for opioid studies as it was used in six of the 12 reptilian species assessed. Although these nociception methods are validated (Chrisman et al., 1997; Couture É et al., 2017; Dahlin et al., 2012; Greenacre et al., 2006; Kanui et al., 1990; Le Bars et al., 2001; Williams et al., 2016), not all are representative. Thermal stimulus tests may not be representative of analgesia reptiles as they have diminished responses to noxious heat stimuli compared to mammals due to their ectothermic nature (Mader, 2008; Makau et al., 2021). Snakes in particular respond less to thermal stimuli as they have a smaller proportion of thermoreceptors, due to the presence of more mechanoreceptors (Mader, 2006). Thermal stimulus tests may also not be representative of other types of nociceptive pain, such as fractures or surgery, due to different nociceptive pathways being triggered when thermoreceptors and other nociceptors are activated (Smith & Lewin, 2009). Therefore, although validated, nociceptive tests may not be representative.

In this review, the NSAID data was mainly extracted from case reports due to a lack of analgesic studies of NSAIDs performed on reptiles. Some cases involved injured reptiles with varying extents of severity, which may have affected the reptiles’ reactions to NSAIDs in the case reports. On the other hand, in the opioid pharmacodynamic studies, animals tested were all healthy, thus health status was unlikely a factor affecting the animals’ response to the nociceptive tests. Additionally, experimental temperature is an important variable that should be controlled due to the ectothermic nature of reptiles. A reptile with body temperature out of its optimal temperature range may react less optimally to nociceptive tests compared to reptiles with temperature within their optimal body temperature range (Reid, 2018). Therefore, comparison of results from these tests may not be accurate since data from studies of reptiles with body temperature out of its optimal temperature range may not be optimal. Since most experimental temperatures of the studies collated were within the reptiles’ POTZ, temperature is unlikely a factor that affects the accuracy and interpretation of results.

## 5 Pharmacokinetic Data

An understanding of the pharmacokinetic parameters of NSAIDs and opioids in the reptilian species studied would enable one to appreciate the extent of intra- and inter-species variability in drug disposition, and subsequently, aid safe dose extrapolation.

Appendix Table 6 summarises the reptilian species in which pharmacokinetic studies on NSAIDs and opioids were performed. Studies were conducted mainly in squamates and chelonians, with no studies found in crocodilians. The pharmacokinetics of both NSAIDs and opioids were studied in loggerhead sea turtles, red-eared slider turtles, and yellow-bellied slider turtles. Only NSAIDs were studied in green iguanas, kemp's ridley sea turtles, green sea turtles, hawksbill turtles, and eastern box turtles, while only opioids were studied in bearded dragons, ball pythons, and prehensile-tailed skinks.

Table 8 summarises key pharmacokinetic parameters obtained from studies of meloxicam, ketoprofen, tolfenamic acid and ketorolac in reptiles. Table 9 summarises key pharmacokinetic parameters obtained from studies of buprenorphine, fentanyl, hydromorphone, tapentadol and tramadol in reptiles.

**Table 8:** Summary of pharmacokinetic parameters from studies of meloxicam, ketoprofen, tolfenamic acid and ketorolac in reptiles.

**Species**	**Dosage regimen**	**CL (ml/h/kg)**	**t_1/2_ (h)**	**V (L/kg)**	**MRT (h)**	**Reference**	
**MELOXICAM**							
Loggerhead sea turtles	IV 0.1 mg/kg single dose	5.52 ± 3.52	38.5 ± 42.58	0.17 ± 0.04 0.19 ± 0.02^c^	63.92 ± 70.64	(Lai et al., 2015)	
	IM 0.1 mg/kg single dose	N.R.	3.26 ± 2.78	N.R.	4.51 ± 3.68	(Lai et al., 2015)	
	SC 2 mg/kg single dose	N.R.	2.99 ± 2.00	N.R.	4.24 ± 1.89	(Norton et al., 2021)^e^	
Red-eared slider turtles	IV 0.2 mg/kg single dose	18.00 ± 2.32	9.78 ± 2.23	0.22 ± 0.03^c^	N.R.	(Uney et al., 2016)	
	IV 0.22^x^ mg/kg	19	7.57	0.18^c^	N.R.	(Rojo-Solis et al., 2009)	
	IM 0.2 mg/kg single dose	N.R.	13.53 ± 1.95	N.R.	N.R.	(Uney et al., 2016)	
	IM 0.5^x^ mg/kg	-	-	-	-	(Rojo-Solis et al., 2009)	
	PO 0.5^x^ mg/kg	-	-	-	-	(Rojo-Solis et al., 2009)	
Yellow-bellied sliders	IM 0.2 mg/kg single dose	N.R.	4.17 ± 1.25	N.R.	N.R.	(Di Salvo et al., 2016)	
	IC 0.2 mg/kg single dose	N.R.	5.73 ± 3.11	N.R.	N.R.	(Di Salvo et al., 2016)	
	PO 0.2 mg/kg single dose	N.R.	15.20 ± 5.51	N.R.	N.R.	(Di Salvo et al., 2016)	
Green iguanas	IV 0.2 mg/kg single dose	37.17 ± 16.08	9.93 ± 4.92^d^	0.49 ± 0.27	14.30 ± 5.72	(Divers et al., 2010)	
	PO 0.2 mg/kg single dose	40.17 ± 10.35	12.96 ± 8.05^d^	0.75 ± 0.48	26.63 ± 10.05	(Divers et al., 2010)	
**KETOPROFEN**							
Loggerhead sea turtles*	IV 2 mg/kg single dose	10.00 ± 0.00^a^	3.60 ± 0.28^a^	0.07 ± 0.00	5.19 ± 0.41	(Thompson et al., 2018)^e^	
		40.00 ± 10.00^b^	2.12 ± 0.18^b^	0.11 ± 0.03	3.06 ± 0.25		
	IM 2 mg/kg single dose	20.00 ± 0.00^a^	2.83 ± 0.41^a^	0.09 ± 10.00	N.R.		
		50.00 ± 10.00^b^	1.93 ± 0.37^b^	0.13 ± 0.02	N.R.		
Green iguanas	IV 2 mg/kg single dose	67	31.00^d^	1.20^c^	18	(Tuttle et al., 2006)	
	IM 2 mg/kg single dose	N.R.	8.30^d^	N.R.	8.2		
**TOLFENAMIC ACID**							
Red-eared slider turtles	IV 2 mg/kg single dose	14.47 ± 0.55	17.55 ± 0.40	0.30 ± 0.01^g^	20.79 ± 0.51	138.38 ± 5.22	(Corum et al., 2019)
	IV 4 mg/kg single dose	13.26 ± 0.40	20.39 ± 0.97	0.31 ± 0.02^g^	23.12 ± 1.09	301.86 ± 8.93	(Corum et al., 2019)
	IM 2 mg/kg single dose	N.R.	22.49 ± 2.07	N.R.	28.06 ± 1.03	155.88 ± 9.03	(Corum et al., 2019)
Green sea turtles	IV 4 mg/kg single dose	1.00 ± 0.00	32.76 ± 4.68	0.03 ± 0.01	53.84 ± 2.80	7378.08 ± 1028.79	(Raweewan et al., 2020a)
	IM 4 mg/kg single dose	N.R.	53.69 ± 3.38	N.R.	61.99 ± 3.51	5313.36 ± 755.08	(Raweewan et al., 2020a)
Hawksbill turtles	IV 4 mg/kg single dose	1.20 ± 0.40	38.92 ± 6.31	0.079 ± 0.02	37.10 ± 3.79	3088.49 ± 356.44	(Raweewan et al., 2020b)
	IM 4 mg/kg single dose	N.R.	41.09 ± 9.32	N.R.	44.07 ± 3.96	2999.56 ± 586.50	(Raweewan et al., 2020b)
**KETOROLAC**							
Loggerhead sea turtles*	IM 0.25 mg/kg single dose	0.1	11.87	1.46	14.68	-	(Gregory et al., 2021)^f^
Eastern box turtles⁎⁎	IM 0.25 mg/kg single dose	0.02	9.78	0.26^h^	10.37	-	(Cerreta et al., 2019)

Note: all values for intramuscular administration presented are calculated as CL/F and V/F, and all volume of distribution values presented as apparent V, unless otherwise stated. Note: values are rounded up to 2 decimal points. IV, intravenous; IM, intramuscular; SC, subcutaneous; IC, intracoelomic; CL, clearance; t_1/2, elimination half-life; V, volume of distribution; MRT, mean residence time; AUC_0-∞_, area under the plasma concentration versus time curve from dosing to infinity; N.R., not reported._

a values attained via individual analysis of S-isomer of ketoprofen

b values attained via individual analysis of R-isomer of ketoprofen

c apparent Vss

d terminal half-life

e study used juvenile turtles, which may affect pharmacokinetic values.

f study used juvenile turtles, which may affect pharmacokinetic values.

g apparent Vss

⁎ healthy juvenile turtles

⁎⁎ adult turtles suffering from traumatic injury

**Table 9:** Summary of pharmacokinetic parameters from studies of buprenorphine, fentanyl, hydromorphone, tapentadol, and tramadol in reptiles.

**Species**	**Dosage regimen**	**C_max_ (ng/ml)**	**AUC_0-_**_∞_**(ng.h/ml)**	**CL (ml/h/kg)**	**V (L/kg)**	**t_1/2_ (h)**	**Reference**
**BUPRENORPHINE**							
Red-eared slider turtle	SC (FL) 0.02mg/kg	151.4±158.8	320.1±281.7	108.4±76.0	0.13^a^	45.3±21.6	(Kummrow et al., 2008)
	SC (FL) 0.05mg/kg	549.2±442.6	978.3±570.2	84.8±69.5	0.09^a^	35.4±44.4	(Kummrow et al., 2008)
	SC (HL) 0.02mg/kg	29.0±26.0	-	-	0.69^a^	-	(Kummrow et al., 2008)
**FENTANYL**							
Ball python	TD 12μg/h	14.7	-	516	-	-	(Darrow et al., 2016)
Prehensile-tailed skink	TD 2.5μg/h	1.55±0.98	65.45±48.34	3768±2807	131.9±81.5	16.25±10.7	(Gamble, 2008)
**HYDROMORPHONE**							
Bearded dragon	SC 0.5mg/kg	141.7±67.0	747±790	669.34*	3.53^a^	2.54±1.60	(Hawkins et al., 2019)
	SC 1mg/kg	368.8±137.5	1819±1369	549.75*	2.71^a^	3.05±1.80	(Hawkins et al., 2019)
Red-eared slider turtle	SC 0.5mg/kg	1396±2160	1957±2609	255.49*	0.36^a^	2.96±3.87	(Hawkins et al., 2019)
	SC 1mg/kg	4829±3557	5686±4245	175.87*	0.21^a^	2.06±0.94	(Hawkins et al., 2019)
**TAPENTADOL**							
Red-eared slider turtle	IM 5mg/kg	1899±242	8987±4879	55200±39600	4.65±1.34	5.22±2.98	(Giorgi et al., 2014)
Yellow-bellied slider turtle	IM 5mg/kg	1641±749	7773±5751	63600±48000	4.30±1.79	4.01±2.10	(Giorgi et al., 2015a)
**TRAMADOL**							
Loggerhead sea turtle	PO 5mg/kg	373±153	18211±12928	275.56*	13.40^a^	20.35±21.52	(Norton et al., 2015)
	PO 10mg/kg	719±331	45038±37758	222.03*	13.91^a^	22.67±23.72	(Norton et al., 2015)
Yellow-bellied slider turtle	IM (FL) 10mg/kg	58220±25180	398500±119750	34.70±11.16	1.25±0.29	28.70±15.77	(Giorgi et al., 2015b)
	IM (HL) 10mg/kg	14930±3320	450440±126000	27.38±8.00	1.55±0.34	50.33±18.21	(Giorgi et al., 2015b)

SC, subcutaneous injection; TD, transdermal patch; IM, intramuscular injection; PO, per oral; FL, injection administered in the forelimb; HL, injection administered in the hindlimb; C_max_, peak plasma drug concentration; AUC_0-∞_, area under the plasma concentration versus time curve from dosing to infinity; CL, clearance; V, volume of distribution; t_1/2_, elimination half-life; ^a^ value calculated using other pharmacokinetic parameters reported and rounded off to 2 decimal places.

### 5.1 Similarities and Differences in Half-Life

The t_1/2_ of a drug depends on both primary pharmacokinetic parameters, CL and V. If the disposition of drug follows linear kinetics, t_1/2_ should not differ when given via different routes of administration. However, t_1/2_ may differ between species due to differences in CL or V.

#### 5.1.1 Half-Life Differs Between Routes of Administration

Since t_1/2_ is expected to remain constant for drugs following linear kinetics despite dosing via different routes of administration, it was important to note that certain trends observed contrasted these expected observations. The half-life of meloxicam, which exhibits linear kinetics (Information, 2022o), was longer when dosed orally (PO) compared to intramuscular (IM), intravenous (IV) and intracoelomic (ICo) routes in yellow-bellied slider turtles (Table 8). Additionally, the t_1/2_ of meloxicam was also longer when dosed PO compared to IV in green iguanas (Table 8). The disposition of meloxicam is probably absorption-rate limited, which resulted in the longer half-life. This suggests that less frequent dosing may be required when meloxicam is dosed orally in these two species.

Secondly, t_1/2_ of meloxicam was about four-fold longer in loggerhead sea turtles compared to red-eared slider turtles when dosed IV (Lai et al., 2015; Uney et al., 2016) (Table 8). This may be due to lower CL in loggerhead sea turtles than the red-eared sliders, but similar volume of distribution at steady state (V_ss_) in both species (Lai et al., 2015; Rojo-Solis et al., 2009). However, the trend was reversed when meloxicam was dosed IM. This suggests that interspecies pharmacokinetic trends may vary significantly with different routes of administration.

Lastly, t_1/2_ of tramadol was two-fold higher with hindlimb compared to forelimb administration in yellow-bellied slider turtles (Giorgi et al., 2015b) (Table 9). This may be due to the possibility that injection sites may affect the pharmacokinetics of drugs, a point of consideration that has been highlighted and discussed in previous literature (Holz et al., 1997a; Kummrow et al., 2008; Yaw et al., 2018). Caudal blood from hindlimb administration drains mainly to the liver in turtles. Since tramadol (Lewis & Han, 1997) undergoes extensive first-pass metabolism, caudal administration results in higher drug metabolism. However further research on the reason for this observation is required as the argument explains that injections sites may affect drug disposition but does not explain reasons for a longer t_1/2_.

Therefore, routes of administration should be considered when dose extrapolating between various drug administration methods in reptiles as they may result in varied drug pharmacokinetics. Mechanistic studies to elucidate the cause for the differing half-life of the same drug administered via different routes should be conducted to understand the underlying cause to illuminate this observation.

#### 5.1.2 Half-Life Trends are Species-Specific

It was observed that t_1/2_ of tolfenamic acid was significantly longer after both IM and IV dosing in green sea turtles and hawksbill turtles compared to red-eared slider turtles (Table 8). This may be due to the approximately 13-fold and 11-fold higher CL, or the 10-fold and 4-fold larger V observed in red-eared sliders compared to green sea turtles and hawksbill turtles, respectively, after IV administration of tolfenamic acid. This trend is further supported by the significantly longer MRT observed in green sea turtles and hawksbill turtles (Table 8). Another observation was that the t_1/2_ of IV meloxicam was four-fold longer in loggerhead sea turtles compared to red-eared slider turtles (Lai et al., 2015; Uney et al., 2016) (Table 8). This may be due to the three times higher CL in red-eared sliders than loggerhead sea turtles. These observations suggest that t_1/2_ of tolfenamic acid and meloxicam may be shorter in red-eared slider turtles compared to other chelonians, indicating faster drug elimination and possibly more frequent dosing in red-eared slider turtles. However, it is crucial to note that there are anomalies to this trend. For example, red-eared slider turtles showed a three-fold longer t_1/2_ of IM meloxicam than yellow-bellied slider turtles (Di Salvo et al., 2016; Uney et al., 2016) (Table 8).

Despite these differences, similar trends in t_1/2_ were also observed. When tramadol was dosed orally in loggerhead sea turtles and IM in yellow-bellied slider turtles, t_1/2_ values were similar (Giorgi et al., 2015b; Norton et al., 2015) (Table 9). This may possibly be due to similar fold change of CL and V between loggerhead sea turtles and yellow-bellied slider turtles.

The inconsistent observation of half-life trends across different species and drugs provides evidence that dose extrapolation between species may not be safe and species-specific studies should be conducted for individual drugs.

#### 5.1.3 Comparing Half-Life Trends between Reptiles and Mammals

Since the disposition of NSAIDs and opioids are well-characterized in mammals, a further step was undertaken to analyze how the disposition of these drugs compare. The half-life of tolfenamic acid after both IV and IM administration in red-eared slider turtles was approximately two-fold longer than that of meloxicam (Corum et al., 2019; Rojo-Solis et al., 2009; Uney et al., 2016) (Table 8). This may be due to the relatively lower CL observed in the red-eared sliders after tolfenamic acid administration. However, this contradicts mammalian data which shows t_1/2_ of meloxicam (t_1/2_ = 20h) (Türck et al., 1996) is longer than that of tolfenamic acid (t_1/2_ 8.01-13.40h) (Lees et al., 1998). Additionally, apart from t_1/2_ of ketoprofen being similar in reptiles and mammals (t_1/2_ = 0.4-3.5h) (Papich, 2021), the other NSAIDs showed very different t_1/2_ values in these two orders. The half-life of meloxicam in mammals is longer than that in reptiles, while t_1/2_ of tolfenamic acid and ketorolac are shorter in mammals than reptiles.

From the opioid data, t_1/2_ of buprenorphine (Kummrow et al., 2008), fentanyl (Gamble, 2008) and tramadol (Giorgi et al., 2015b; Norton et al., 2015) are significantly longer than that of hydromorphone (Hawkins et al., 2019) and tapentadol (Giorgi et al., 2015a; Giorgi et al., 2014) (Table 9). However, this is not congruent with mammalian data which shows that the t_1/2_ of buprenorphine (t_1/2_ = 3-5h) (Inturrisi, 2002), fentanyl (t_1/2_ = 3.7h) (Inturrisi, 2002), tramadol (t_1/2_ = 5-6h) (Beakley et al., 2015), hydromorphone (t_1/2_ = 2-3h) (Inturrisi, 2002) and tapentadol (t_1/2_ = 3.93h) (Terlinden et al., 2007) are similar. Additionally, only the t_1/2_ of hydromorphone and tapentadol are similar in reptiles and mammals. T_1/2_ of buprenorphine, fentanyl and tramadol are much longer in reptiles than mammals.

This subsection further highlights the importance of conducting pharmacokinetic studies in each individual species as the extensive knowledge and understanding of disposition of drugs gathered from mammalian studies may not be applicable to reptiles.

### 5.2 Similarities and Differences in Clearance

The elimination of drugs can be via metabolism or excretion. Clearance is a primary pharmacokinetic parameter describing elimination of drugs from the body. Hepatic clearance is dependent on physiological variables such as blood flow, drug-protein binding and intrinsic clearance as described by the well-stirred model (Pang & Rowland, 1977). On the other hand, renal clearance would depend on blood flow, drug-protein binding and possibly urine flow and pH, depending on the characteristic of the drug and whether it is largely filtered, secreted and/or reabsorbed.

#### 5.2.1 Clearance Trends are Species-Specific

The CL of IM ketorolac, a hepatically metabolized drug (Buckley & Brogden, 1990), was observed to be five-fold higher in loggerhead sea turtles compared to eastern box turtles (Cerreta et al., 2019; Gregory et al., 2021) (Table 8). However, it was intriguing to note that despite this, the t_1/2_ and MRT observed in the loggerhead sea turtles compared to eastern box turtles were approximately 1.2- and 1.4-fold longer, respectively. One possible explanation is that loggerhead sea turtles had a 5.6-fold larger V than eastern box turtles. However, as there was only data on V in loggerhead sea turtles and apparent V_ss_ in eastern box turtles, the calculated fold change in V may not be accurate.

Another observation was the exceptionally low CL of tolfenamic acid, also hepatically metabolized (Pedersen, 1994), observed in green sea turtles and hawksbill turtles which was less than 2 mL/kg/h (Raweewan et al., 2020a; Raweewan et al., 2020b) (Table 8). This was further supported by the long t_1/2_ that exceeded 24 hours for both species, and the significantly longer MRT observed in both species compared to the red-eared slider turtles. This suggests that the green sea turtles and hawksbill turtles may have an inherently lower hepatic metabolism than other chelonians, and hence may require less frequent dosing. However, more data is required to confirm this species-specific CL trend.

From the opioid data, it was observed that the CL of fentanyl, another hepatically metabolized drug (Trescot et al., 2008), was very different between skinks and ball pythons (Darrow et al., 2016; Gamble, 2008) (Table 9). This raises the question of whether drug disposition may be significantly different between lizard and snake species. However, similar CL has been observed between geckos (Agius et al., 2020), bearded dragons (Salvadori et al., 2017) and rattlesnakes (Waxman et al., 2015) in non-opioid drugs such as enrofloxacin. Thus, whether drug CL differs within the squamates likely depends on the drug of interest.

#### 5.2.2 Clearance Trends Between Squamates and Chelonians

It was observed that the CL of meloxicam and ketoprofen, both hepatically metabolized (Bethesda, 2012; Chesné et al., 1998), were significantly higher in green iguanas compared to red-eared slider turtles and loggerhead sea turtles (Table 8). CL of meloxicam in green iguanas was two-fold and seven-fold higher than red-eared slider turtles and loggerhead sea turtles, respectively (Divers et al., 2010; Lai et al., 2015; Uney et al., 2016). CL of IV ketoprofen was up to six-fold higher in green iguanas than loggerhead sea turtles (Thompson et al., 2018; Tuttle et al., 2006). However, the study of ketoprofen in loggerhead sea turtles analyzed individual stereoisomers, which may not be an accurate comparison with the CL values in green iguanas.

From the opioid data, the calculated apparent CL of hydromorphone, a hepatically metabolized drug (Trescot et al., 2008), was about three-fold higher in bearded dragons compared to red-eared slider turtles (Hawkins et al., 2019) (Table 9). Therefore, these trends highlight the possibility that CL of drugs that are largely hepatically metabolized may be higher in squamates compared to chelonians. Further research of the CL of other analgesics in a greater number of squamates and chelonians could provide greater insight to determine whether this trend is indeed a true trend observed between squamates and chelonians.

Overall, ketorolac, tolfenamic acid, meloxicam, ketoprofen, fentanyl and hydromorphone are mainly metabolized in the liver. Therefore, species differences in CL may be due to differences in hepatic metabolism.

### 5.3 Differences in C_max_ and AUC_0-∞_

It was observed that the AUC_0-∞_ of IV tolfenamic acid in hawksbill turtles and green sea turtles was 10-fold and 24-fold higher than red-eared sliders, respectively for the same dose administered (Corum et al., 2019; Raweewan et al., 2020a; Raweewan et al., 2020b) (Table 8). This may be due to a significantly higher CL of tolfenamic acid in red-eared slider turtles than hawksbill and green sea turtles.

Another observation was that the C_max_ of buprenorphine and tramadol (Giorgi et al., 2015b; Kummrow et al., 2008) (Table 9) were lower with hindlimb administration compared to forelimb administration in turtles for the same dose administered. This supports the argument that injection sites may affect the pharmacokinetics of drugs which has been discussed previously. Since buprenorphine (Elkader & Sproule, 2005) and tramadol (Lewis & Han, 1997) undergo extensive first-pass metabolism, caudal administration results in higher drug metabolism. This leads to less drug entering systemic circulation and hence a lower C_max­_.

Additionally, C_max_ and AUC_0-∞_ values of hydromorphone studied on reptiles were unexpected (Table 9). Given the same dose of 0.5mg/kg administered subcutaneously, the AUC_0-∞_ of hydromorphone was three-fold higher in red-eared slider turtles than bearded dragons, even though the t_1/2_ was similar (Hawkins et al., 2019). One postulation is that red-eared slider turtles have a higher proportion of subcutaneous fat, serving as a reservoir of hydromorphone. Thus, they experience higher drug exposure due to higher bioavailability from this depot effect. However, the exact reason for varied C_max_ and AUC_0-∞_ is unknown and warrants further investigation.

In summary, the pharmacokinetic trends of NSAIDs and opioids are drug- and species-specific. Although there were differences, there were also drug examples where the pharmacokinetic parameters were similar across chelonians. For example, tapentadol was tested in red-eared slider turtles (Giorgi et al., 2014) and yellow-bellied slider turtles (Giorgi et al., 2015a) while tramadol was tested in yellow-bellied slider turtles (Giorgi et al., 2015b) and loggerhead sea turtles (Norton et al., 2015) (Table 9). Despite being tested in different species, pharmacokinetic parameters of tapentadol and t_1/2_ of tramadol were similar in the two species studied. Overall, it is worth noting that despite the differing drug disposition, most of the NSAIDs and opioids appeared safe and effective in the species studied. However, the interactions between the pharmacodynamics and pharmacokinetics of NSAIDs and opioids should be investigated further.

### 5.5 Critical Analysis on the Design of Pharmacokinetic Studies

As many pharmacokinetic studies tested various dosing regimens in the same animal, the washout period is a component that must be considered. If the washout period is insufficient, residual drug in the animals’ systemic circulation may affect the pharmacokinetics of the next dosing regimen tested. As such, the washout period should be at least five half-lives (t_1/2_) long, the minimal time needed for a drug to be considered mostly eliminated from the body. For example, t_1/2_ of tapentadol in yellow-bellied slider turtles was reported to be six hours (Giorgi et al., 2015a). An interval of at least 30 hours is required, hence the washout period of one month was sufficient. Based on calculations using t_1/2_ in reptiles and humans, which was used when studies did not report reptilian t_1/2_, all washout periods in the studies included in this review were considered sufficient. However, as t_1/2_ may differ between reptiles and humans, washout periods should only be compared with reptilian t_1/2_ for a more accurate conclusion on its sufficiency. Additionally, some studies only screened a subset of animals to determine if the washout period was sufficient. This may be inaccurate as data may not be representative of the entire population, and the washout period may not actually be sufficient in all the animals tested.

For drugs administered via subcutaneous and intramuscular routes, the site of injection is another component that must be considered. Circulation from the caudal region empties into both the renal portal system and liver. In reptiles, blood drains mainly to the kidney in snakes but drains mainly to the liver in lizards and chelonians (Holz et al., 1997b). Therefore, it is likely that injection site would have a significant effect on renally eliminated drugs in snakes, while injection site would have a significant effect on hepatically eliminated drugs in lizards and chelonians. As such, since NSAIDs and opioids are mainly hepatically eliminated (Day et al., 1988; Trescot et al., 2008), they should theoretically be injected cranially in lizards and chelonians, to prevent any variation in drug pharmacokinetics. This is supported by the evidence that showed that t_1/2_ of tramadol differs with hindlimb compared to forelimb administration in yellow-bellied slider turtles (Table 9).

## 6 Conclusion

Reptilian dosing is often extrapolated from mammalian dosing as there is a lack of pharmacological studies of NSAIDs and opioids in reptiles. Therefore, this report aimed to evaluate the analgesic effects of NSAIDs and opioids on reptiles through collating evidence from pharmacological studies of NSAIDs and opioids on reptiles and analysing the pharmacodynamic and pharmacokinetic observations to derive any possible trends that may aid safe and efficacious use of the drugs in different reptile species

Most of the existing reports of NSAID used in reptiles did not observe any adverse effects directly associated to the respective NSAID use, with meloxicam being the most well-studied. Despite the current absence of analgesic efficacy studies for NSAIDs in reptiles, most reports observed behavioural improvements in reptiles after NSAID treatment. Morphine and fentanyl were studied in the greatest number of reptilian species with analgesic effects observed with the doses used, while side effects such as sedation were observed most with butorphanol use.

It was observed that drug pharmacokinetic trends tend to be drug- and species-specific. One interesting trend observed was that drug t_1/2_ tends to differ between different routes of drug administration. Of greatest significance was the t_1/2_ of IV meloxicam that was four-fold higher in loggerhead sea turtles compared to red-eared slider turtles, but the trend was reversed when meloxicam was dosed IM (Lai et al., 2015; Uney et al., 2016). Another unique trend observed was that CL of drugs may be higher in squamates compared to chelonians. Of greatest significance was the CL of meloxicam and hydromorphone, which was seven-fold higher in green iguanas (Divers et al., 2010) and three-fold higher in bearded dragons (Hawkins et al., 2019) than loggerhead sea turtles (Lai et al., 2015) and red-eared slider turtles (Hawkins et al., 2019) respectively.

Generally, there is a lack of pharmacokinetic studies performed in reptiles, with only 14 NSAID studies and eight out of the 21 opioid studies examined in this review reporting pharmacokinetic data. It would thus be optimal if individual pharmacodynamic and pharmacokinetic studies can be performed for each species, to provide a more holistic assessment of the analgesic efficacy of NSAIDs and opioids in reptiles. Additionally, many reptilian species are understudied, especially the crocodilians with only one study being performed on crocodiles in opioids and none in NSAIDs. In squamates and chelonians, most opioid studies were performed on ball pythons and red-eared slider turtles, respectively, while most NSAID studies were performed on loggerhead sea turtles. Few studies were performed on other species. Therefore, further studies involving a wider range of reptilian species should be conducted to ensure a more balanced view of the analgesic efficacy of NSAIDs and opioids in reptiles.

Furthermore, the expression of COX enzymes in reptiles are only known for yellow-bellied geckos (Buch et al., 2018; Buch et al., 2017), eastern box turtles (Royal et al., 2012) and ball pythons (Sadler et al., 2016), while the types of opioid receptors present in reptiles are only known for freshwater turtles (Xia & Haddad, 2001) and ball pythons (Kharbush et al., 2017). Mechanistic studies to identify COX enzyme expression and opioid receptors and investigate the interactions between the NSAID and opioid drugs and the various COX enzymes and opioid receptors in reptiles should be performed to aid more accurate drug selection for treatment.

While general trends that are drug- or species-specific could not be drawn from this critical review, we found several interesting trends and conflicting findings that warrant further investigation. Furthermore, the evidence collated provided a birds’ eye view on which drug and species were most well-studied and which are lacking evidence for their use. In conclusion, there is a dire need for more studies to be conducted to understand the complex pharmacokinetic-pharmacodynamic interactions of various drugs in more reptilian species to ensure safe and efficacious use of these drugs in alleviating pain for the animals.

## Ethical statement

This is a review of current literature and no live animals were involved.

## Declaration of Competing Interest

The authors declare that they have no known competing financial interests or personal relationships that could have appeared to influence the work reported in this paper.

## Figures and Tables

**Table A1:** Keywords and Search Terms Used in Review Search Strategy.

**Research Subtopic**	**Keywords**	**Related Search Terms**
Reptile species	Reptile	Reptile, Reptilia, Reptilian
Chelonia	Chelonia, Testudines, Turtle, Tortoise, Terrapin, Slider	Turtle, Tortoise, Terrapin, Sea Turtle, Red-eared slider, Yellow-bellied slider, Black-bellied slider, Loggerhead sea turtle, Speke's hinged tortoise, Snapper, Cooter, Leatherback, Chelonia, Chelonian, Pleurodirans, Cryptodirans
Squamata	Squamata, Lizard, Snake	Lizard, Snake, Chameleon, Gecko, Iguana, Gila monster, Serpentes, Bearded dragon, Corn snake, Green Iguana, Komodo dragon, Slow worm, Slowworm, Prehensile-tailed skink, Cobra, Viperidae, Elapids, Pythoninae, Mamba, Python, Boa, Boidae, Boinae, Viper, Skink, Lacertilia, Amphisbaenia, Diploglossa, Gekkota, Iguania, Platynota, Scincomorpha, Alethinophidia, Colubrid, Scolecophidia, Agamid, Anoles, Varanid, Tegu
Crocodilian	Crocodilian, Alligator, Crocodile	Alligator, Crocodile, Crocodilian, Caiman, Cayman, Crocodylidae, Gharial, Freshwater crocodile
NSAIDs	Non-steroidal anti-inflammatory drug	Anti-inflammatory agents, anti-inflammatory, Non-steroidal, Meloxicam, Flunixin, Carprofen, Tolfenamic acid, Ketoprofen, Ketorolac
Opioids	Opioid	Buprenorphine, Butorphanol, Etorphine, Fentanyl, Hydromorphone, Methadone, Meperidine, Morphine, Oxycodone, Oxymorphone, Pethidine, Tapentadol, Tramadol
Physiology	Physiology, Physiological	Physiological characteristic, Metabolic, Metabolic rate, Basal metabolic rate, Caudal, Cranial, Posterior, Anterior, Drug effect, Renal, Renal portal system, Kidney, Hepatic, Hepatic portal system, Liver, Ectotherm, Ectothermic, Poikilotherm, Poikilothermic, Body weight, Body size, Weight, Size, Ambient temperature, Body temperature, Temperature
Pharmacology	Pharmacology, Pharmacological, Pharmacokinetic, Pharmacodynamic	AUC, Area under curve, Clearance, Bioavailability, Half-life, Elimination half-life, Absorption half-life, Absorption, Distribution, Metabolism, Excretion, Elimination, Disposition, Drug absorption, Drug distribution, Drug metabolism, Drug excretion, Drug elimination, Drug disposition, Drug clearance, Injection site, Site of injection, Administration route, Route of administration
Adverse effects	Adverse, Effect	Adverse effects, Effect, Side effect, Unwanted effects, Unwanted, ADR, Detrimental effects, Detrimental, Drug effects, Adverse drug effects, Unwanted drug effects, Adverse drug reaction, Drug reaction
Pan management	Pain	Pain indicator, Indicator of pain, Pain indication, Pain management, Management, Analgesia, Analgesic, Nociception, Nociceptive, Nociceptive assay, Antinociception, Antinociceptive, Tail flick, Tail-flick, Hot-plate, Hot plate, Hargreaves Assay, Von Frey Assay, Formalin Assay, Formalin test, Capsaicin test, Capsaicin assay, Vet, Veterinary
COX expression	COX, expression	Cyclooxygenase, COX, COX-1, COX-2, COX-3, cyclo-oxygenase, COX1, COX2, COX3, Cyclo-Oxygenase I, Cyclo Oxygenase I, Prostaglandin H Synthase-1, Prostaglandin H Synthase 1, Prostaglandin Synthase, COX-1 Prostaglandin, Prostaglandin H Synthase-2, PTGS2, PTGHS-2, PTGS1, PTGHS-1

**Table A2:** Summary of range of doses for each reptile species from studies of meloxicam, flunixin, ketorolac, etoricoxib, ketoprofen, carprofen, tolfenamic acid on reptiles.

**NSAID**	**Dose range [Route(s) of administration] (mg/kg unless otherwise stated)**												
	**Yellow-bellied gecko**	**Green iguana**	**Monitor** **lizard**	**Komodo** **dragon**	**Bearded** **dragon**	**Ball** **python**	**Eastern** **box turtle**	**Yellow-bellied** **slider**	**Logger-head** **sea turtle**	**Red-eared** **slider**	**Kemp's ridley sea turtle**	**Green sea turtle**	**Hawksbill turtle**
Meloxicam	-	0.2 [IV, IM] 0.2 – 5 [PO]	-	10.5 mg [PO]	0.2 [IM]	0.3 [IM]	-	-	0.1 [IV, IM] 1 [SC]	0.2 [IV, IM]	1 [SC]	1 [SC]	-
Flunixin	-	-	0.5 [IM]	-	-	-	-			-	-	1 [IM]	-
Ketorolac	-	-	-	-	-	-	0.25 [IM]	-	0.25 [IM]		-	-	-
Etoricoxib	25 [PO]	-	-	-	-	-	-	-	-	-	-	-	-
Ketoprofen	-	-	-	-	-	-	-		2 [IV, IM]	-	-	-	-
Carprofen	-	2 [IM]	-	-	-	-	-	-	-	-	-	-	-
Tolfenamic acid	-	-	-	-	-	-	-	-	-	2 – 4 [IV] 2 [IM]	-	4 [IV, IM]	4 [IV, IM]

PO, by mouth; IM, intramuscular injection; SC, subcutaneous injection; IV, intravenous injection; NSAID, non-steroidal anti-inflammatory drugs.

**Table A3:** Summary of range of doses for each reptile species from studies of buprenorphine, butorphanol, fentanyl, hydromorphone, morphine, pethidine, tapentadol and tramadol on reptiles.

**Opioid**	**Dose range [Route(s) of administration] (mg/kg unless otherwise stated)**											
	**Crocodile**	**Ball python**	**Bearded dragon**	**Corn snake**	**Green iguana**	**Prehensile-tailed skink**	**Tegus**	**Black-bellied slider turtle**	**Logger-head sea turtle**	**Red-eared slider turtle**	**Speke's hinged tortoise**	**Yellow-bellied slider turtle**
Buprenorphine	-	-	-	-	0.02 – 0.1 [IM]	-	-	-	-	0.02 – 1 [SC]	-	-
Butorphanol	-	5 – 10 [IM]	2 – 20 [SC]	2 – 20 [SC]	0.4 – 8 [IM]	-	5 – 10 [IM]	-	-	2.8 – 28 [SC]	-	-
Fentanyl	-	3 – 12 μg/h [TD]	-	-	-	2.5μg/h [TD]	-	0.05 [SC]	-	0.05 [SC]	-	-
Hydromorphone	-	-	0.5 – 1 [SC]	-	-	-	-	-	-	0.5 – 1 [SC]	-	-
Morphine	0.05 – 1 (Ventafridda et al.)	10 [IM]	1 – 20 [SC]	1 – 40 [SC]	0.4 – 1 [IM]	-	5 – 10 [IM]	-	-	1.5 – 6.5 [SC]	5 – 20 [ICo]	-
Pethidine	1 – 8 (Ventafridda et al.)	-	-	-	-	-	-	-	-	-	10 – 50 [ICo]	-
Tapentadol	-	-	-	-	-	-	-	-	-	5 [IM]	-	5 [IM]
Tramadol	-	-	-	-	-	-	-	-	5 – 10 [PO]	1 – 25 [PO] 10 – 25 [SC]	-	10 [IM]

PO, by mouth; IM, intramuscular injection; SC, subcutaneous injection; TD, transdermal patch; ICo, intracoelomic injection; IP, intraperitoneal injection.

**Table A4:** Overview of the efficacy observed in studies of buprenorphine, butorphanol, fentanyl, hydromorphone, morphine, pethidine, tapentadol and tramadol on reptiles.

**Opioid**	**Buprenorphine**	**Butorphanol**	**Fentanyl**	**Hydromorphone**	**Morphine**	**Pethidine**	**Tapentadol**	**Tramadol**
Crocodile	-	-	-	-	✓ (Kanui & Hole, 1992)	✓ (Kanui & Hole, 1992)	-	-
Ball python	-	✗ (Olesen et al., 2008; Williams et al., 2016)	✓ (Darrow et al., 2016; Kharbush et al., 2017)	-	✗ (Williams et al., 2016)	-	-	-
Bearded dragon	-	✗ (Sladky et al., 2008)	-	✓ (Hawkins et al., 2019)	✓ (Sladky et al., 2008)	-	-	-
Corn snake	-	✓ (Sladky et al., 2008)	-	-	✗ (Sladky et al., 2008)	-	-	-
Green iguana	✗ (Greenacre et al., 2006)	✓ (Greenacre et al., 2006)	-	-	✓ (Greenacre et al., 2006)	-	-	-
Prehensile-tailed skink	-	-	✓ (Gamble, 2008)	-	-	-	-	-
Tegus	-	✗ (Leal et al., 2017)	-	-	✓ (Leal et al., 2017)	-	-	-
Black-bellied slider turtle	-	-	✓ (Kaminishi et al., 2019)	-	-	-	-	-
Loggerhead sea turtle	-	-	-	-	-	-	-	✓ (Norton et al., 2015)
Red-eared slider turtle	✓ (Kummrow et al., 2008; Mans et al., 2012)	✓ (Kinney et al., 2011; Sladky et al., 2007)	✓ (Kaminishi et al., 2019)	✓ (Hawkins et al., 2019; Kummrow et al., 2008)	✓ (Kinney et al., 2011; Sladky et al., 2007)	-	✓ (Giorgi et al., 2014)	✓ (Baker et al., 2011)
Speke's hinged tortoise	-	-	-	-	✓ (Wambugu et al., 2010)	✓ (Wambugu et al., 2010)	-	-
Yellow-bellied slider turtle	-	-	-	-	-	-	✓ (Giorgi et al., 2015a)	✓ (Giorgi et al., 2015b)

✗, no analgesic efficacy observed with any dosage regimen tested; ✓, analgesic efficacy observed with at least one dosage regimen tested; -, no analgesic study found.

**Table A5:** Overview of the adverse effects observed in studies of buprenorphine, butorphanol, fentanyl, hydromorphone, morphine, pethidine, tapentadol and tramadol on reptiles.

**Opioid**	**Buprenorphine**	**Butorphanol**	**Fentanyl**	**Hydromorphone**	**Morphine**	**Pethidine**	**Tapentadol**	**Tramadol**
Crocodile	-	-	-	-	N.R. (Kanui & Hole, 1992)	N.R. (Kanui & Hole, 1992)	-	-
Ball python	-	✓ (Olesen et al., 2008; Williams et al., 2016)	✓ (Darrow et al., 2016; Kharbush et al., 2017)	-	✗ (Williams et al., 2016)	-	-	-
Bearded dragon	-	N.R. (Sladky et al., 2008)	-	✓ (Hawkins et al., 2019)	N.R. (Sladky et al., 2008)	-	-	-
Corn snake	-	N.R. (Sladky et al., 2008)	-	-	N.R. (Sladky et al., 2008)	-	-	-
Green iguana	✗ (Greenacre et al., 2006)	✓ (Greenacre et al., 2006)	-	-	✗ (Greenacre et al., 2006)	-	-	-
Prehensile-tailed skink	-	-	✗ (Gamble, 2008)	-	-	-	-	-
Tegus	-	N.R. (Leal et al., 2017)	-	-	N.R. (Leal et al., 2017)	-	-	-
Black-bellied slider turtle	-	-	✗ (Kaminishi et al., 2019)	-	-	-	-	-
Loggerhead sea turtle	-	-	-	-	-	-	-	✗ (Norton et al., 2015)
Red-eared slider turtle	✓ (Kummrow et al., 2008; Mans et al., 2012)	✓ (Kinney et al., 2011; Sladky et al., 2007)	✗ (Kaminishi et al., 2019)	✗ (Hawkins et al., 2019; Kummrow et al., 2008)	✓ (Kinney et al., 2011; Sladky et al., 2007)	-	✓ (Giorgi et al., 2014)	✓ (Baker et al., 2011)
Speke's hinged tortoise	-	-	-	-	N.R. (Wambugu et al., 2010)	N.R. (Wambugu et al., 2010)	-	-
Yellow-bellied slider turtle	-	-	-	-	-	-	✓ (Giorgi et al., 2015a)	✗ (Giorgi et al., 2015b)

✗, no adverse effect observed with any dosage regimen tested; ✓, adverse effect observed with at least one dosage regimen tested; N.R., adverse effect not reported; -, no study of adverse effects found.

**Table A6:** Summary of reptilian species in which pharmacokinetic studies on NSAIDs and opioids were performed.

**Drug**	**Squamata**				**Chelonia**						
	Green iguana	Bearded dragon	Prehensile-tailed skink	Ball python	Loggerhead sea turtle	Kemp's ridley sea turtle	Green sea turtle	Red-eared slider turtle	Yellow-bellied slider turtle	Hawksbill turtle	Eastern box turtle
**NSAIDs**											
Meloxicam	✓	-	-	-	✓	✓	✓	✓	✓	-	-
Ketoprofen	✓	-	-	-	✓	-	-	-	-	-	-
Tolfenamic acid	-	-	-	-	-	-	✓	✓	-	✓	-
Ketorolac	-	-	-	-	✓	-	-	-	-	-	✓
**Opioids**											
Buprenorphine	-	-	-	-	-	-	-	✓	-	-	-
Fentanyl	-	-	✓	✓	-	-	-	-	-	-	-
Hydromorphone	-	✓	-	-	-	-	-	✓	-	-	-
Tapentadol	-	-	-	-	-	-	-	✓	✓	-	-
Tramadol	-	-	-	-	✓	-	-	-	✓	-	-

✓Pharmacokinetic study available in specific reptile species; -, no pharmacokinetic study found in specific reptile species.

## Appendix

Table A1Table A2, Table A3, Table A4, Table A5, Table A6
